# B-Lines Lung Ultrasonography Simulation Using Finite Element Method

**DOI:** 10.3390/diagnostics12112751

**Published:** 2022-11-10

**Authors:** Fellipe Allevato Martins da Silva, Eduardo Moreno, Wagner Coelho de Albuquerque Pereira

**Affiliations:** 1 Engineering Program-COPPE, Federal University of Rio de Janeiro, Rio de Janeiro 21941-901, Brazil; 2 Departamento Física Aplicada, Instituto de Cibernetica Matematica y Fisica-ICIMAF, Habana 10400, Cuba

**Keywords:** lung ultrasonography, B-lines, simulation, COMSOL, finite element method

## Abstract

Introduction: Lung Ultrasonography (LUS) is a fast technique for the diagnosis of patients with respiratory syndromes. B-lines are seen in response to signal reverberations and amplifications into sites with peripheral lung fluid concentration or septal thickening. Mathematical models are commonly applied in biomedicine to predict biological responses to specific signal parameters. Objective: This study proposes a Finite-Element numerical model to simulate radio frequency ultrasonic lines propagated from normal and infiltrated lung structures. For tissue medium, a randomized inhomogeneous data method was used. The simulation implemented in COMSOL^®^ used Acoustic Pressure and Time-Explicit models, which are based on the discontinuous Galerkin method (dG). Results: The RF signals, processed in MATLAB^®^, resulted in images of horizontal A-lines and vertical B-lines, which were reasonably similar to real images. Discussion: The use of inhomogeneous materials in the model was good enough to simulate the scattering response, similar to others in the literature. The model is useful to study the impact of the lung infiltration characteristics on the appearance of LUS images.

## 1. Introduction

Lung Ultrasonography (LUS) is a fast technique for the diagnosis of patients with respiratory syndromes. It is an exam that allows for the evaluation of disease development and response to treatment [1,2,3]. Pulmonary illnesses with a slow evolution of worsening symptoms cannot be monitored properly by chest X-rays [4,5]. LUS appears to be a reliable bedside exam with good sensitivity to detect pulmonary congestion, cardiogenic pulmonary edema, pneumonia, interstitial lung syndrome (e.g., systemic sclerosis disease), and consolidation [4,6,7]. In the past, lung ultrasound imaging was thought to be useless as a clinical tool due to the high acoustic impedance mismatch between the air inside the lungs and the chest wall tissues [8,9].

**Figure 1:**
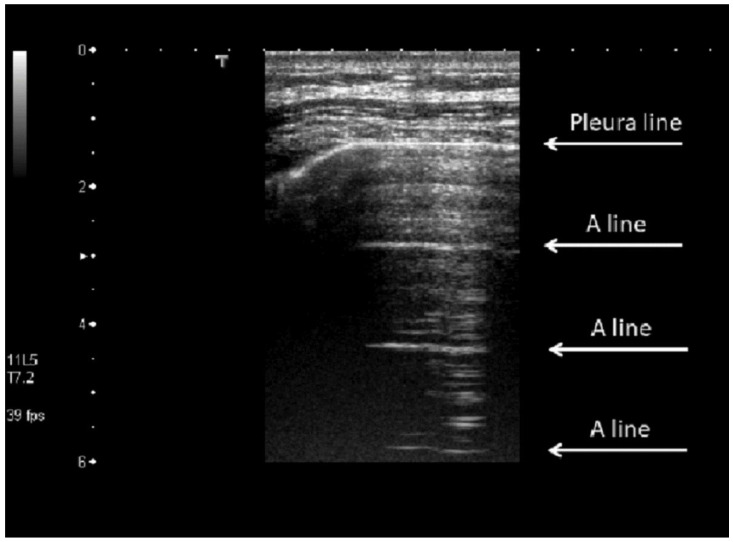
A-lines in a healthy lung. The top image presents a thoracic wall layer (skin, fat and muscles) that ends with the hyperechoic pleural line. Hyperechoic horizontal lines called A-lines, produced by the wave reverberations, are shown beneath. Reprinted/adapted with permission from [10]. 2022, Acoustical Society of America.

B-mode imaging of the lungs does not reproduce the anatomical structure of the aerated organ, but the image artifact patterns formed in this exam correlate well with specific lung conditions [10]. The acoustic impedance of lungs depends on the air proportion inside of them. For the case of a healthy lung filled with air, this impedance is of the order of 0.17 MRayl [11], producing an impedance mismatch that results in a reflection coefficient around 99% on the interface chest muscle/lung pleura. Therefore, ultrasound does not penetrate a healthy aerated lung and the wave reverberates between the pleural and the transducer faces [9,10]. This physical phenomenon is expressed in the ultrasonography as hyperechoic horizontal lines between two acoustic shadows from the ribs. These artifacts are called A-lines (Figure 1).

In interstitial lung syndrome, pulmonary density can be altered by lung congestive diseases (e.g., lung edema and heart failure) or parenchymal diseases (e.g., pneumonia and systemic sclerosis). LUS changes the A-line patterns to the so-called B-line patterns, which are well-defined vertical hyperechoic lines, and laser beam-like lines that emerge at the pleural level, erasing the A-lines [3,12,13,14]. They are directly correlated to the level of liquid in the lung parenchyma [1,2,15]. The presence of B-lines on the LUS has been correlated with disease severity by several score protocols, including scores for the identification and classification of COVID-19 [6,7,16,17,18,19].

**Figure 2:**
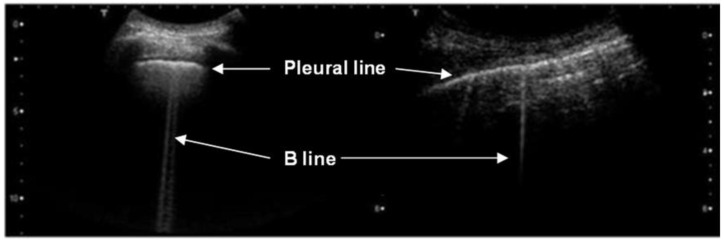
Three examples of B-lines. B-lines are vertical hyperechoic, well-defined and laser beam-like lines that emerge at the pleural level, erasing the A-lines, and move in synchrony with the respiratory cycle. Reprinted/adapted with permission from [10]. 2022, Acoustical Society of America.

The physical explanation of the B-lines’ origin has been the aim of some studies [9,10,20]. The main theory is that B-lines are the consequences of signal reverberation and amplification into the peripheral extravascular concentration of lung fluids, or parenchyma septal thickening. Those places become propagation sites that allow for the penetration of the ultrasonic wave in small marginal parts of the lung tissue (Figure 2) [9,10,21].

Understanding physical phenomena using mathematical models is a commonly applied method in biomedical engineering and similar areas. Ultrasound numerical simulations may be used to infer and predict biological responses to specific signal parameters, and was employed in various studies describing LUS A-line and B-line origins [20,21]. K-wave has been the first-choice software toolbox in LUS simulations to solve the wave equation due to the MATLAB^®^’s familiarity in the research environment [21,22]. However, the commercial software COMSOL^®^ had more complete tools of the Finite Element Method (FEM). Version 5.5 has specific physics tools, known as acoustic time-explicit methods, that allow for pulse propagations to be simulated in a domain with an acoustic array source. These are based on the discontinuous Galerkin method (dG) [23].

The aim of this study was to develop a mathematical model using the Finite Elements Method (FEM) to simulate RF ultrasonic lines propagated from normal and infiltrated lung structures, to emulate LUS image samples with COMSOL^®^ software and MATLAB^®^ signal processing. The numerical study focuses on the physical factors that originate A-lines and B-lines.

## 2. Materials and Methods

### 2.1. Ultrasound Propagation Simulation Techniques

**Figure 3:**
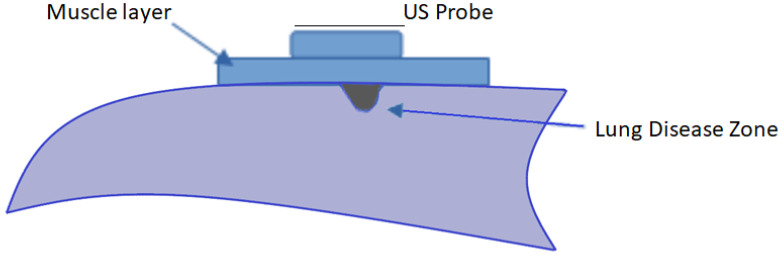
Lung Model Domain. There is a small ellipsoid grey zone of the lung tissue represented as the “lung disease zone” (LDZ).

The first step in FEM simulation was the definition of the structural domain. Figure 3 shows a scheme that represents the ultrasonic array probe over a muscle layer that covers the lung domain. A small ellipsoid grey zone just below the muscle layer represents one part of the lung tissue infiltrated by liquid, named Lung Disease Zone (LDZ).

### 2.2. COMSOL^®^ Models

COMSOL^®^ version 5.5 was used with Acoustic Pressure and Time-Explicit models. These tools used the dG algorithm for the simulation of ultrasonic pulse propagation. This method, compared to the classic acoustic methods, has the advantage of having less degrees of freedom, leading to a shorter processing time. In this paper, a rectangular base of 66 mm in length and 51 mm in height was used.

**Figure 4:**
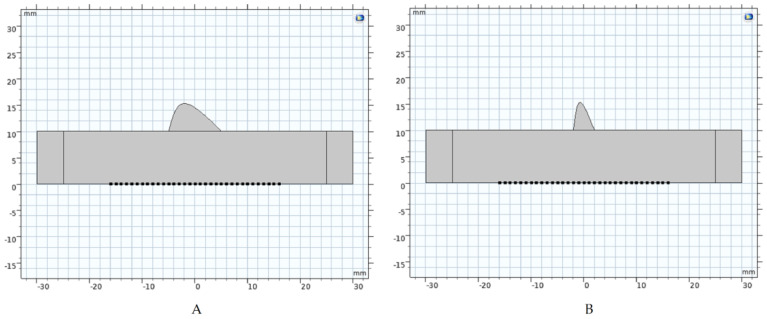
Two COMSOL^®^ domains with 32 array elements (back dots). Domain (**A**) and Domain (**B**). The rectangular area represents the muscle with PML at both left and right sides. The subdomain (in grey) over the rectangle represents the small lung part with inhomogeneity or “Lung disease zone” (LDZ).

In Figure 3, the lung healthy zone assumes the condition of 100% of air, and its acoustic impedance, as mentioned before, is so low (0.17 Mrayl) [6] that only the tissues in the blue zones were taken into consideration in the final COMSOL^®^ domain. Figure 4 shows the domain used for simulation in reversed position (upside down) with respect to Figure 3. In this case, the muscle domain, in a rectangle form, has two perfect matching layer (PML) zones at each lateral side. An ultrasonic linear array with 32 elements (black dots) is shown below. A piece of lung with inhomogeneity is shown at the top and all the area above is lung with 100% air.

The boundary condition in these domains is as follows: 5 mm length and 10 height PML at both sides in the rectangle zone of 50 mm length and 10 mm height. The LDZ was placed in the upper part of the rectangular zone, as an ellipsoid of 5 mm height and two conditions of length: 10 mm (Domain A) and 4 mm (Domain B). A normal sweep velocity with delays is considered for each array element. The array is organized with apertures of five elements, which includes focalization laws at 10 mm in terms of emission. With a sweep step of each five-element group at a time, it is possible to obtain 28 radio frequency (RF) ultrasonic lines.

The superior boundary condition, including the upper part of LDZ, was of Pressure type. Then, it simulates a virtual healthy lung. The other boundaries were “Sound Hard Boundary”. Mesh elements were designed according to the recommendations of maximum size of λ/1.5 [24].

**Figure 5:**
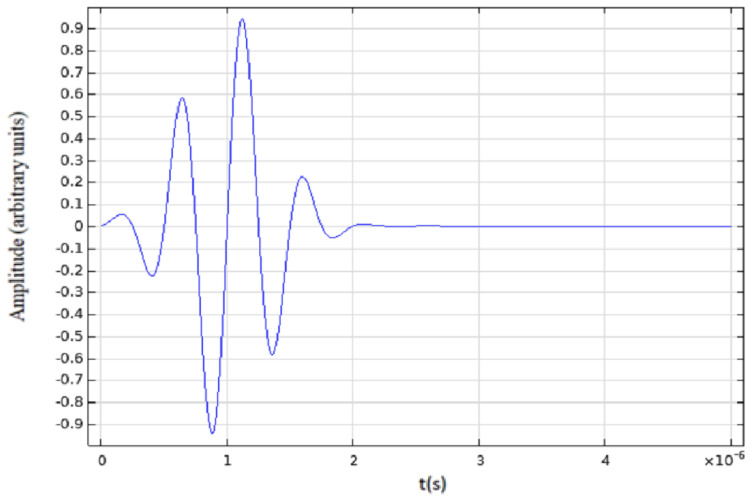
Initial emitted ultrasonic pulse. Sinus function with 2πf_0_t argument modulated by Gaussian envelope Central and frequency f_0_ = 2 MHz.

The initial ultrasonic pulse applied to each element with a central frequency of f_0_ = 2 MHz is shown in Figure 5. The time-domain study was used with a maximum of 50 µs and a step of 0.02 µs. A parametric sweep was necessary to simulate the array emission through 28 steps, where only five elements are allowed for emission each time. The normal sweep velocity mentioned above was made according to the Equation (1):

V(i) = A(i) ∗ pulse(t + B(i)), for i = 1 to 32,(1)

where A(i) = 0 (OFF) or 1 (ON), according to the sweep parameter and the emission organization (five elements ON and the rest OFF). B(i) for each element is the delay at each sweep necessary for focalization in transmission at approximately 10 mm. The function *pulse*, shown in Figure 5, is given by a sinus function with 2πf_0_t argument modulated by Gaussian envelope.

A workstation with 128 GB RAM, 10 TB HDD, and a microprocessor with 40 cores (Intel^®^ Xeon(R) Gold 6230 CPU @ 2.10 GHz × 40) was used. The time performance was around 5 h depending on the material models at 2-MHz ultrasonic frequency.

Average Nonlocal Coupling was defined for each element to obtain the signals (echoes) at each array element. Then, 28 × 32 = 896 RF signals were obtained (each of them with 2500 samples) and exported to a MATLAB^®^ (MathWorks Inc., Massachusetts, EUA) routine [25] where a visualization program displays B-scan images. The images were built using plot algorithms, with a logarithm amplifier applied to each ultrasonic RF signal first, and then a Hilbert transform was used to obtain the signal envelope, which is necessary for composing each imaging line. Each line was made from the RF signal that was produced by the 5-element pulse-echo excitation subgroup, and delay focusing was applied at each receiving signal. An interpolation algorithm was employed to increase the lateral imaging size.

### 2.3. COMSOL^®^ Strategy

**Figure 6:**
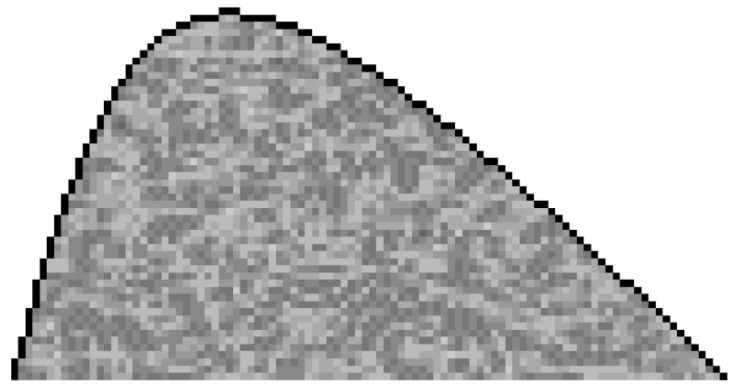
Subdomain distribution function f(x,y) for “lung disease zone” (LDZ). The grey scale indicates the spatial variation in the infiltrated properties of the lung tissue.

For tissue medium, a randomized inhomogeneous data method was used according to Sjodin (2017) [26]. This method assumes that each tissue has a random variation in density and ultrasound velocity. The variation could be modeled by a spatial frequency using a cosine transform [26]. This transform has random phase and amplitude functions with parameters that could be adjusted for each material. To achieve that, a random function f(x,y) with the Results Node functions, in a previous short model of COMSOL^®^ 5.5, was obtained. This was then exported using Data Grid for our Lung COMSOL^®^ model, as an interpolation function to use in the Material Node. Figure 6 shows the case for the lung tissue infiltrated subdomain, where f(x,y) oscillates between ±1 for both velocity and density.

For this domain, the strategy is to define the maximal and minimal values of ultrasonic velocity and density. The maximal value of velocity is 1500 m·s^−1^ and, for density, 1000 kg·m^−3^, which means the lung is modeled as pure water. The minimal values are: vmin = 640 m·s^−1^ and dmin = 430 kg·m^−3^, which corresponds to a lung with 60% of air [11,27]. With these maximal and minimal values, the following equations are obtained for the Material Lung Node for the subdomain of Figure 6:

v(x,y) = 1070 m·s^−1^ + 430 ∗ f(x,y) m·s^−1^ (2)

d(x,y) = 715 kg·m^−3^ + 285 ∗ f(x,y) kg·m^−3^ (3)

These equations ensure the interval for velocity and density, as discussed above. This idea could be extended to other intervals.

**Figure 7:**
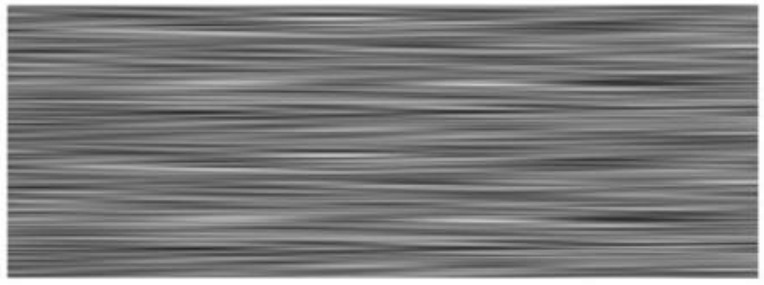
Graphic of the muscle function g(x,y) used on the simulation model. Gray scale indicates the properties of spatial variation for the simulation of muscle fibers.

For the muscle layer, a similar approach was used, with a distribution function g(x,y) obtained as in the previous case, which oscillates between ±1 for both velocity and density. Parameter G defines the variations with values of 0, 5 and 10, for density and velocity, respectively. Figure 7 shows the domain visualization of this function. The distribution is non-symmetric with respect to x–y axes. The idea is to reproduce the muscle fiber structure. Equations (4) and (5) establish the variations in velocity and density for the muscle.

v_muscle_ (x,y)= 1570 m·s^−1^ + G ∗ g(x,y) m·s^−1^ (4)

d_muscle_ (x,y)=1090 kg·m^−3^ + G ∗ g(x,y) kg·m^−3^ (5)

## 3. Results

**Figure 8:**
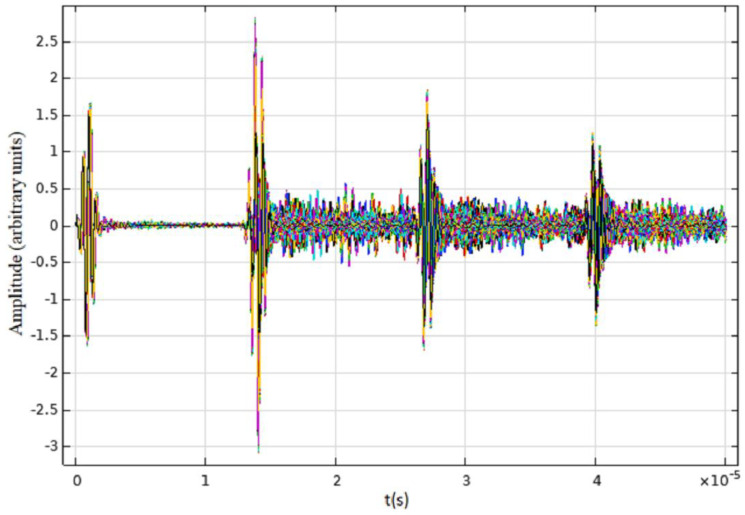
RF signals obtained from COMSOL^®^ corresponding to the domain expressed in Figure 4. The first signal is the emitted pulse, and the others are received echoes.

Figure 8 shows one example of a superimposed RF signal obtained from case A of Figure 4. The initial pulse and three well defined echoes from the far boundary of the muscle can be observed. The random echoes between correspond to the multiple scatterings in the subdomain of the lung disease zone (LDZ).

**Figure 9:**
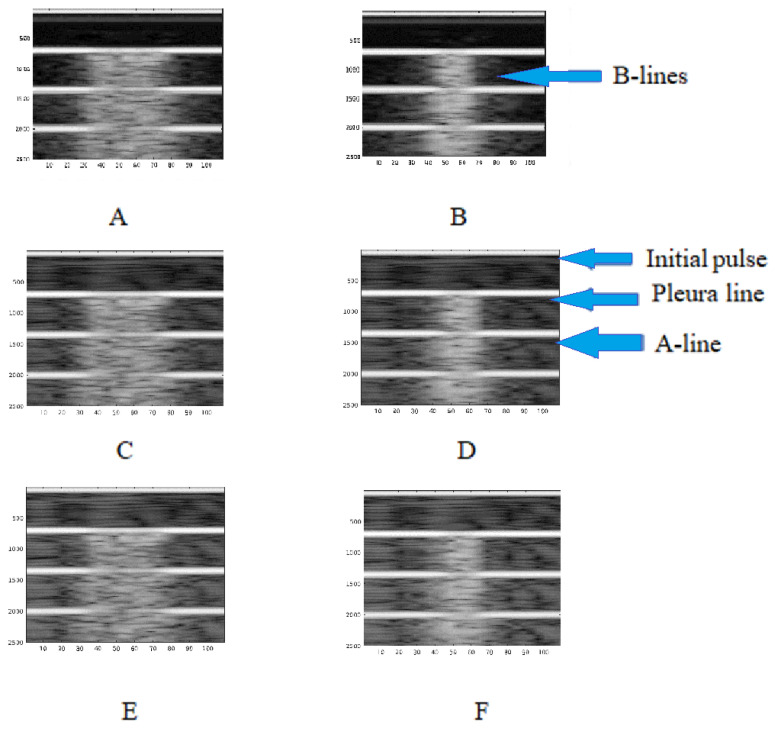
Imaging obtained at different G level and domains. For (**A**) and (**B**), G = 0; (**C**) and (**D**), G = 5; (**E**) and (**F**), G = 10. The left column corresponds to domain A, in Figure 4. The right column corresponds to Domain B in the same figure. The A-lines (horizontal) vs. B-lines (vertical) are shown in B and D, as examples.

The following figure, Figure 9A–F, shows the results of the B-Scan imaging obtained in a MATLAB^®^ routine from the RF COMSOL^®^ signals. Subfigures represent three values of the factor of inhomogeneity G (Equations (4) and (5)) for both domains, according to Figure 4.

The horizontal lines for each image correspond to the initial pulse followed by the echoes (top to bottom). The first echo represents the pleural line, while the second and the others are the named A-lines. These lines correspond to repetitions (reverberations) of the first echo between pleura and transducer, as it is possible to observe the muscle fiber image though several micro-horizontal lines.

Vertical patterns also appeared in every simulation, similar to B-lines. Even though these vertical lines do not make A-lines disappear, they are well defined hyperechoic vertical images rising from the pleural line until the image bottom, as in the clinical LUS exam. It is also possible to observe that the LDZ length is directly correlated with the B-line simulation length.

**Figure 10:**
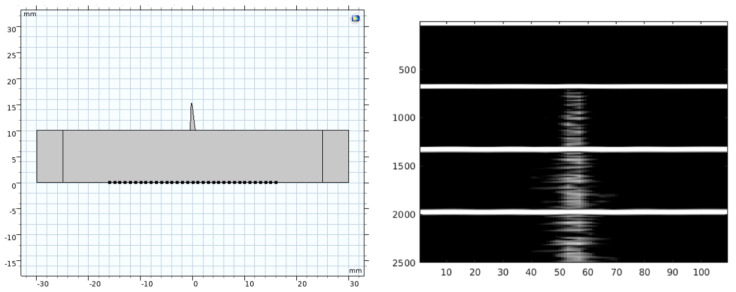
B-Scan imaging obtained at 4 MHz frequency in a new domain with a 1 mm length. Left, it is the simulation domain, and right the image result.

The numerical experiment was also repeated at a frequency of 4 MHz, and with a domain even narrower (1 mm length and 5 mm height). Figure 10 shows the domain and the results with a B-line narrower than the ones obtained previously. This result is more adapted to real medical imaging although it takes about two days to run in our PC computer.

## 4. Discussion

Lung Ultrasonography has become a central focus of study in the literature since it was shown to be a revolutionary way to diagnose and evaluate different types of lung diseases [15,28]. LUS is a fast, precise, real-time and inexpensive exam that allows for health workers to make better choices regarding patient treatment, with the ability to produce high-sensitivity results to detect pulmonary density changes in interstitial syndrome [2,3,12,14,16,29]. While the formation process of A-lines (horizontal image patterns) is well established, the origin of B-lines (vertical laser-like patterns) still need to be better understood. New numerical studies, with more complete lung models, are important to obtain a better understanding of this topic [3,10,21,30].

The origin and explanations of B-lines has been the subject of several studies. Avruch and Cooperberg (1985) [31] were one of the first to describe what was called a “Ring-Down” artifact, a common ultrasound artifact of either a solid streak or parallel bands, associated with gas collection in the intestinal image exam. It is also theorized for lung image artifacts that fluid collection formed by two layers of bubble tetrahedron would guaranty an oscillator site, which amplifies the RF signal and sends it back to the transducer [32]. Demi et al. (2018) [10] deal with a theoretical model based on signal processing and an experimental model of a phantom made by air rods (as bubble drops) inside and agar-agar matrix. Using B-scan instruments, the authors obtained images that simulate the B-lines.

Peschiera et al. (2021) [21] performed numerical simulations using k-wave MATLAB^®^ toolbox to achieve B-lines simulation. The phantom model involved muscle areas, air areas, and aisle areas with muscle and air circle-like areas. B-line images were generated with satisfactory aspects and were analyzed with intensity parameters features. Low frequencies enabled the formation of B-lines in only the largest interalveolar spacing simulation setting. Then, it is assumed that the lower the frequency at which B-lines appear, the larger the channels formed between alveoli, and more severe the lung condition.

Silva et al. (2022) [22] simulated five configurations of LUS phantom using the k-wave MATLAB^®^ toolbox [33]. Phantom simulations were designed with multiple muscle tissue feature circles lined up above multiple circles with air features, representing muscle fibers and pulmonary alveoli, respectively. Circles with water features were placed over the alveoli in five different configurations, and formed B-line images in all designed phantoms. The arrangement with two close water circles was the one with the clearest laser-like B-line.

Formally, COMSOL^®^ uses a library of material properties that cover several human tissues, such as muscle, fat etc. However, all these models assume homogenous media. To simulate a B-line, it is necessary for part of the domain inside the lung to be represented with multiple scatters. This is a very difficult task using the geometry structure of COMSOL^®^, because it implies several boundary conditions with their own meshes (grids).

One possible solution is inhomogeneous material models, as described by Sjodin (2017) [26] for thermal properties. The idea is to use random functions as the coefficients and phase of a cosine transform. Inhomogeneous material models with FEM-dG methods describe basic ultrasound-imaging artifacts in lung diseases, with similar results to other numerical studies [21,22], presenting satisfactory image resolution even when a lower central frequency was employed.

The simulation results of the present study show clear B-lines, as a consequence of LDZ inhomogeneity. The results achieved in this mathematical model were very similar to those of Kameda et al. (2019) [34], who simulated in vitro B-lines with B-mode image acquisition at different RF frequencies (6, 8, 11, and 13 MHz) of a lung phantom made of a spindle-shaped juice sac and glucomannan gel. The inhomogeneity models do not need special geometry components for the COMSOL^®^ models; no complex situation is generated with several boundary conditions, and there is no need for independent meshes. The inhomogeneity material avoids these situations and provides a good modeling and understanding of the multiple scattering in this zone. It has been proposed that this zone acts as a trap for the ultrasonic RF, which reverberates and allows for the LDZ to become a secondary RF source [32].

B-lines are then directly correlated with alterations in the lung zone close to the pleura, as water-like fluids and/or lung parenchyma consolidation [10,21,30]. These results are similar to the ones in the literature, indicating that irregular lung surface may generate B-line patterns [6,8,9,15,16,22,29]. The comprehension of the physical phenomena that generate these artifacts is crucial to categorize the different types of B-lines, and to correlate them with both specific diseases and different stages of diseases [35]. The artifacts’ signal characterization is also crucial to developing quantitative algorithms dedicated to LUS diagnosis and monitoring [3].

The computational processing speed of the LUS simulations was a limitation of this study, as it proved difficult to use central probe frequencies that are commonly employed in clinical exams (e.g., 4 to 7 MHz). Simulation with other frequencies would allow for a better understanding of its correlation with the formation of B-lines. Another limitation is the simplicity of the lung model. Other conditions, such as parenchyma hepatization and calcifications, have yet to be included.

In our work, B-lines were produced by the inhomogeneity of the LDZ (Figure 9 and Figure 10). The use of inhomogeneous materials based on random components in the model was good enough to simulate the scattering response of the tissue. The dG acoustic model is also one good solution to the simulation of ultrasonic pulse propagation for this kind of scenario. Simulation with narrow LDZ gave a more accurate image result, which was closer to the clinical ones.

## 5. Conclusions

A mathematical FEM model using randomized inhomogeneous materials was proposed and developed to simulate an LUS image sample and to evaluate the physical origins of A-line and B-line artifacts. The main mechanism theory of B-line formation is assigned to the reverberation of ultrasonic waves in a layer of lung tissue below the pleura with liquids close to the interfaces that cover the lung. The transversal dimension and structure of these B-lines depends on the structure of the LDZ. In any case, the developed model could be used for multiple LDZ zones and other geometrical considerations. It is also possible to obtain results with different ultrasonic array parameters, such as frequency and dimensions, as well as other possible lung conditions that may generate B-lines; thus, this work does not intend to exhaust this subject.
